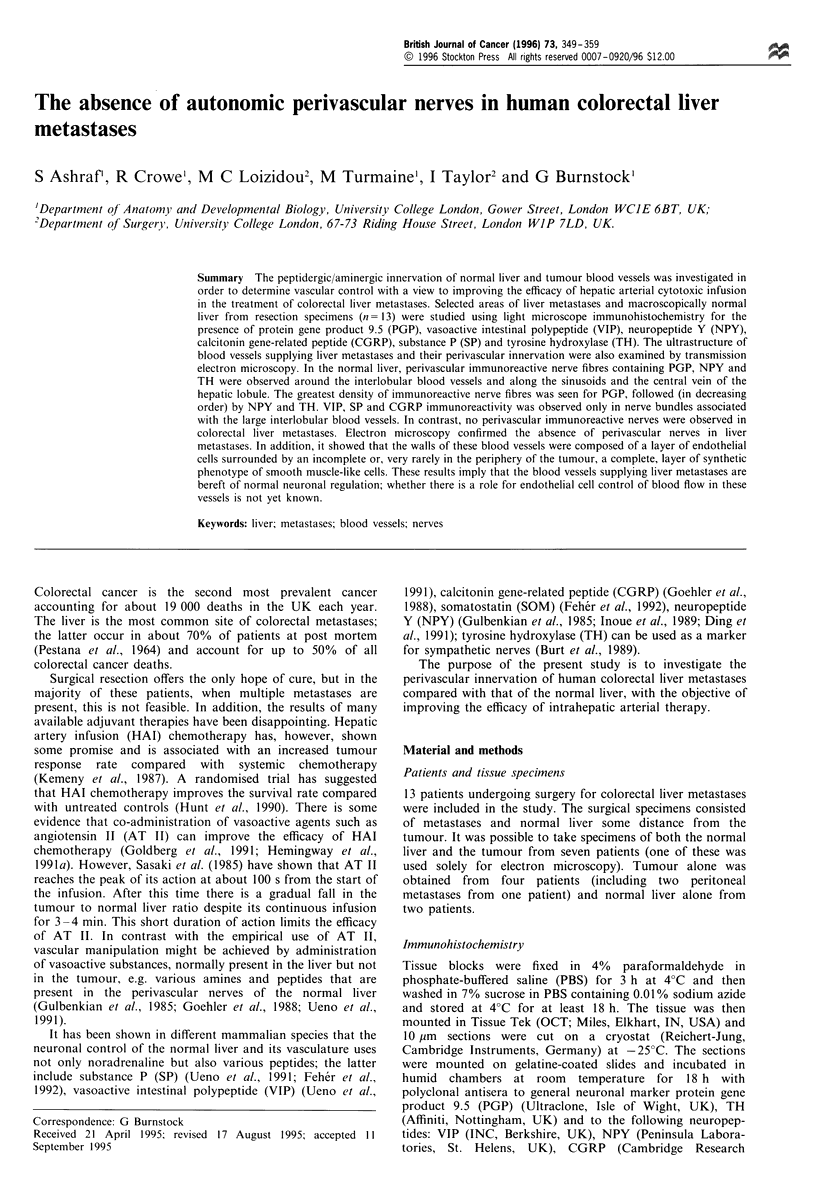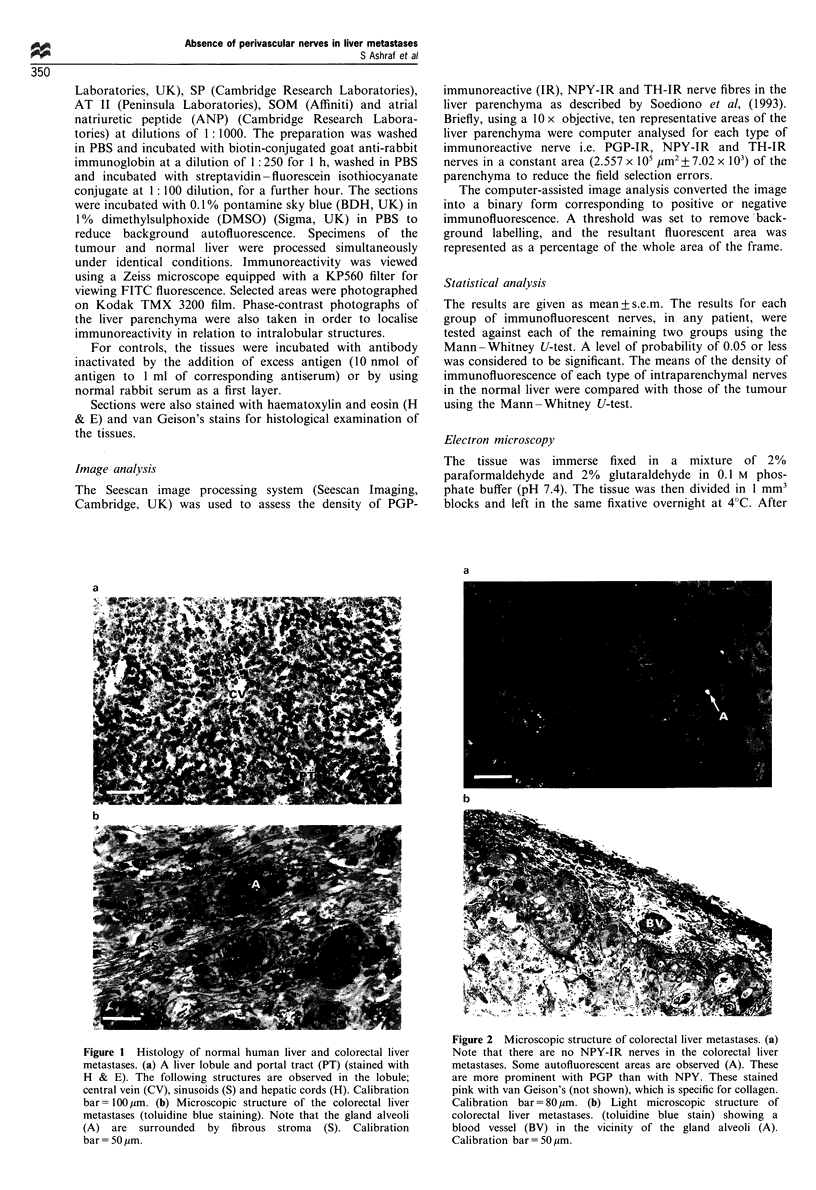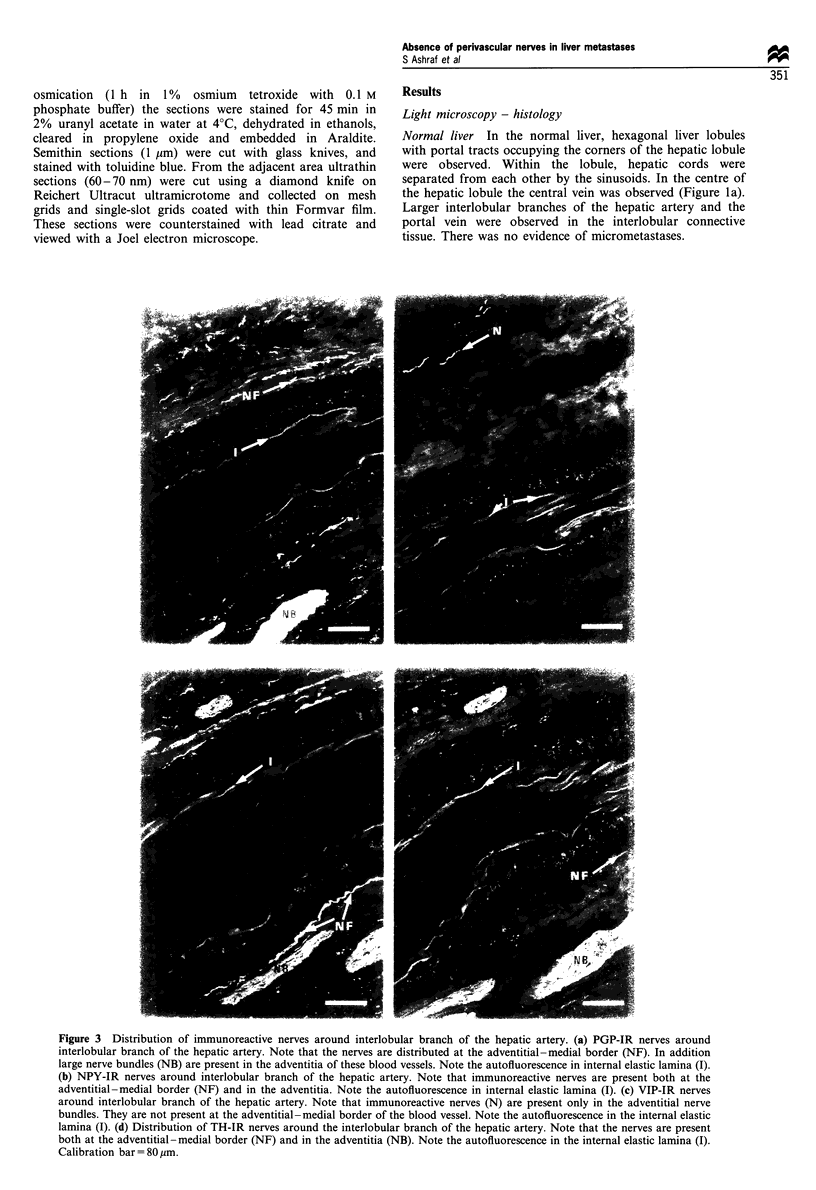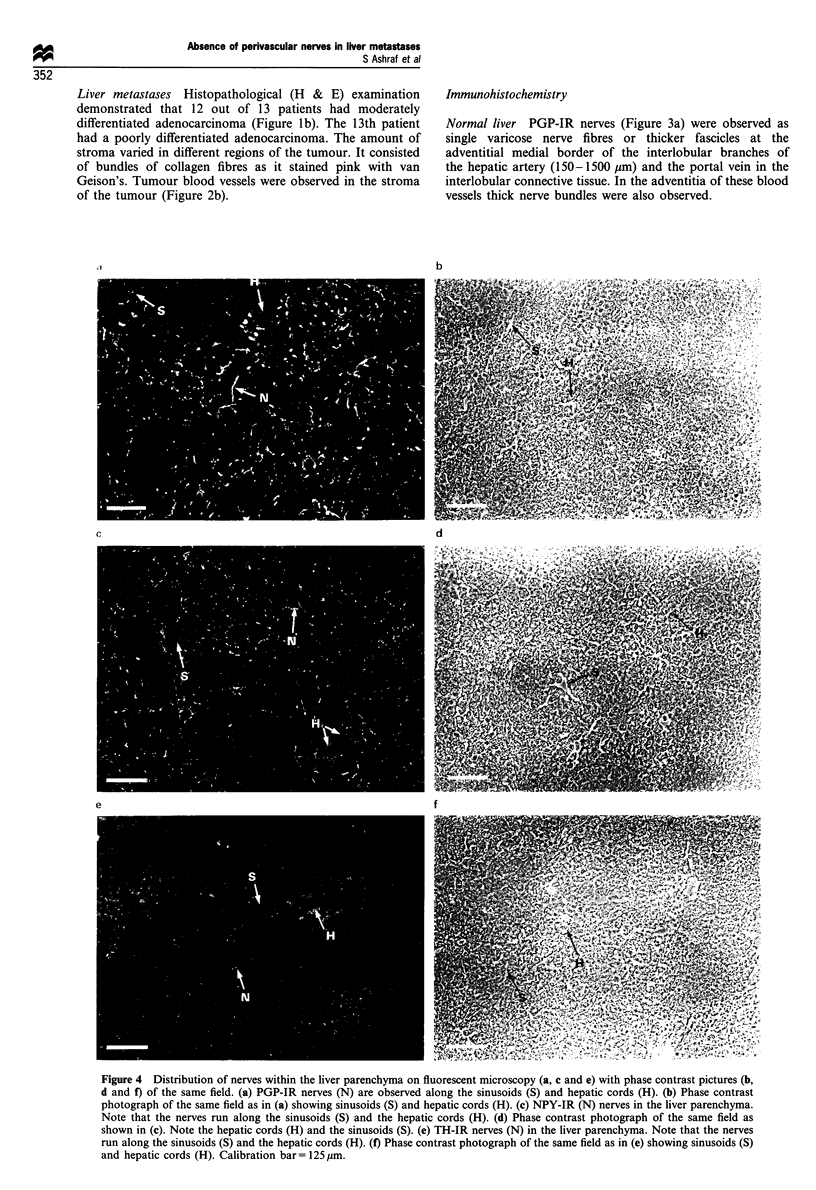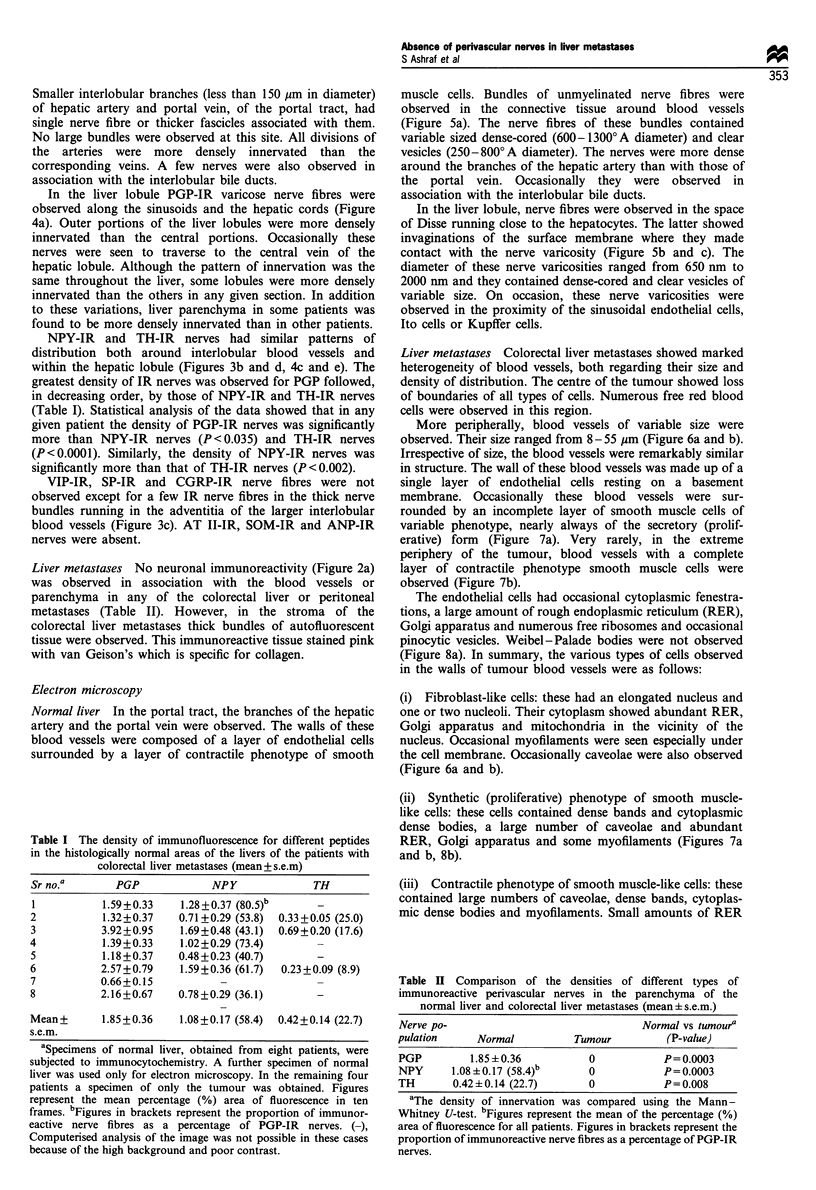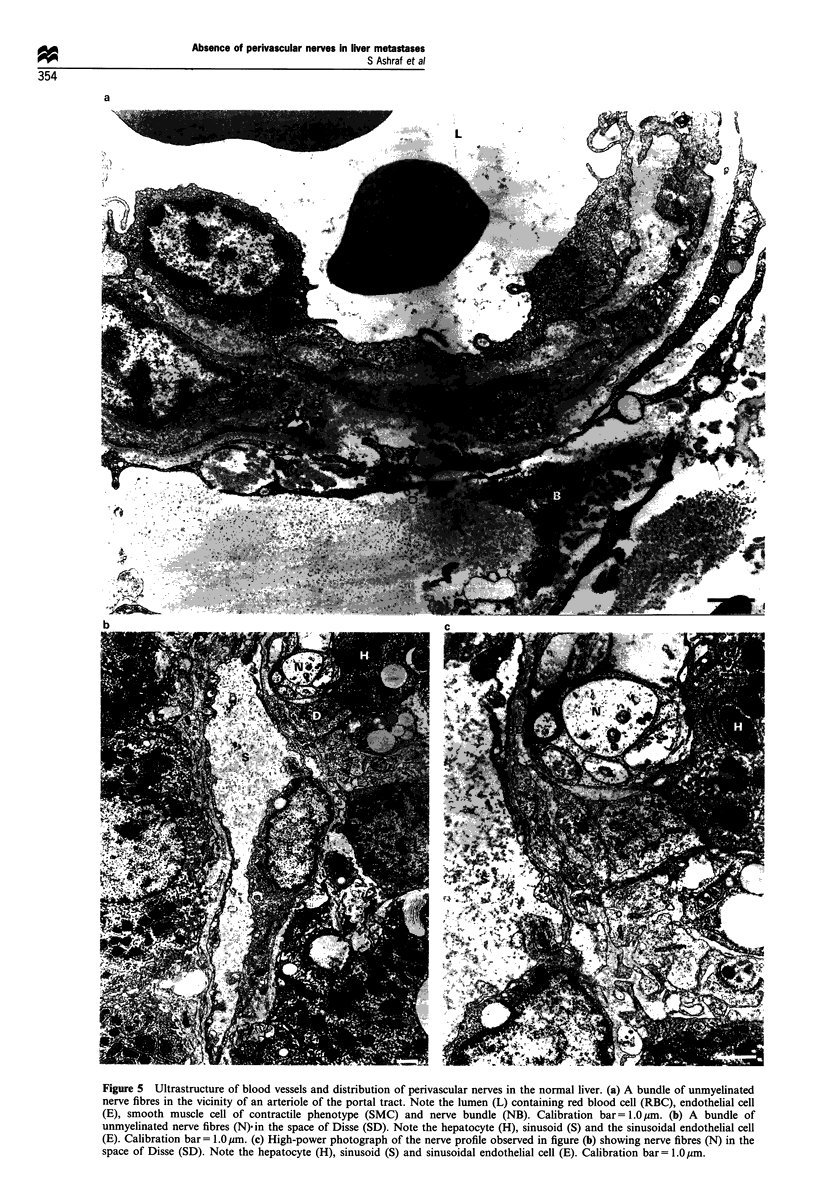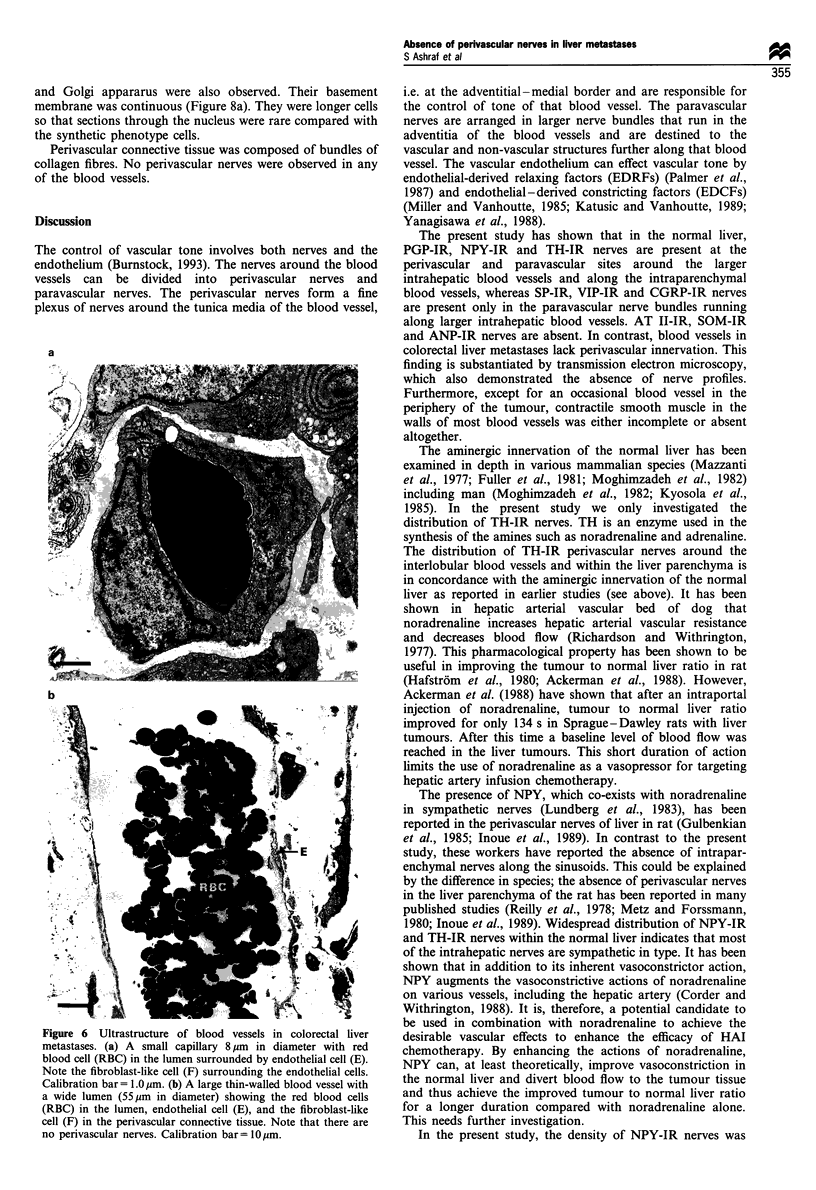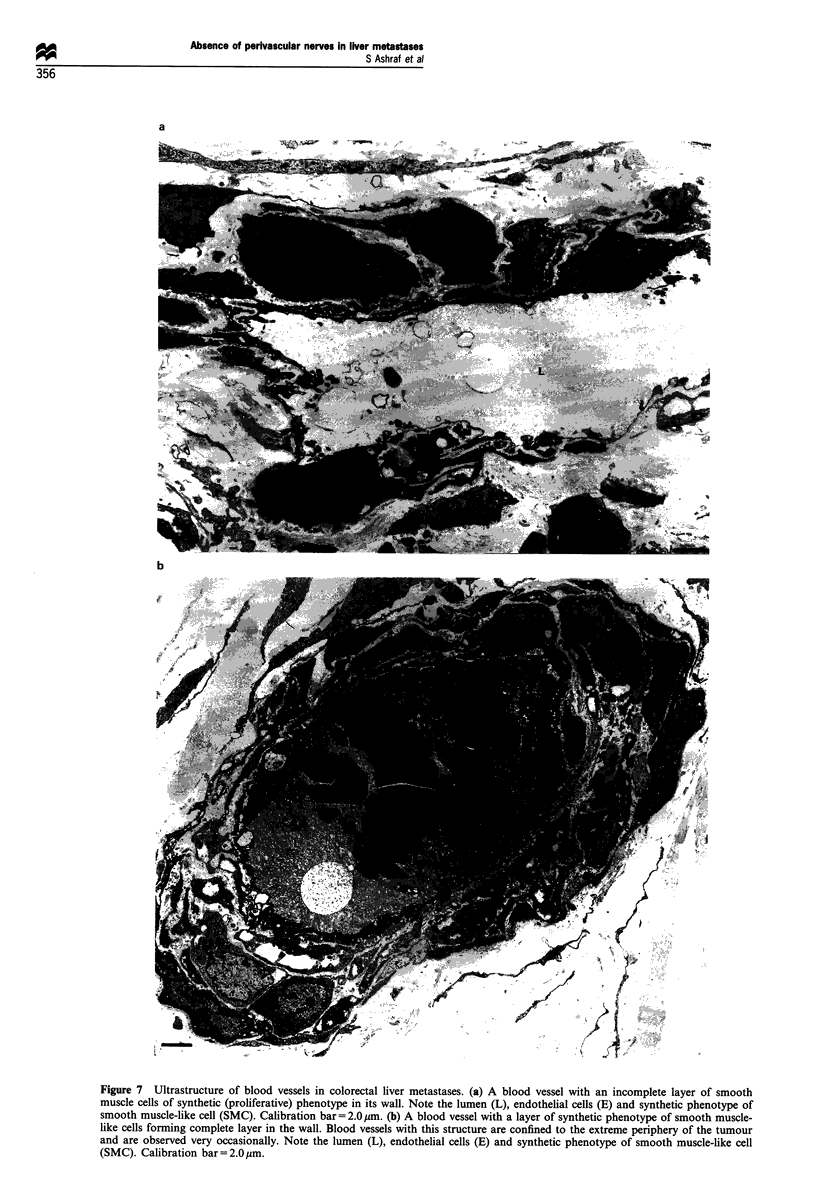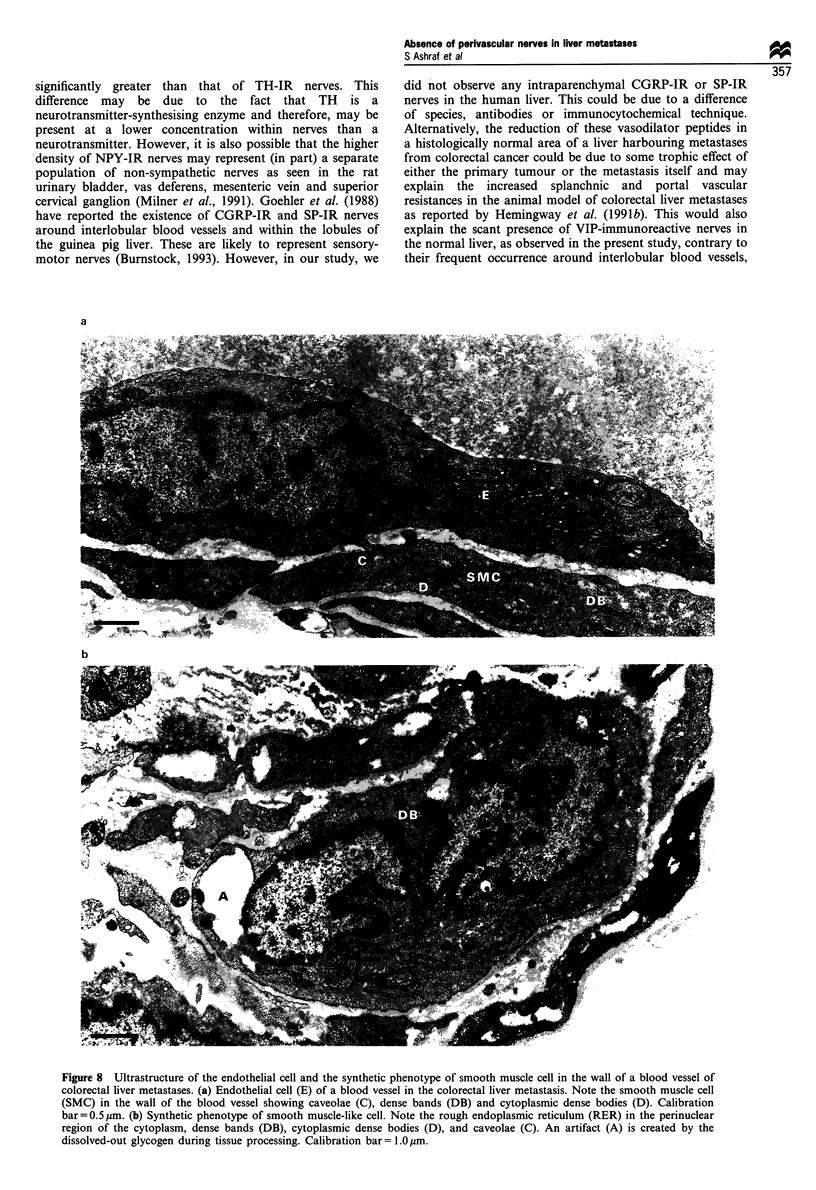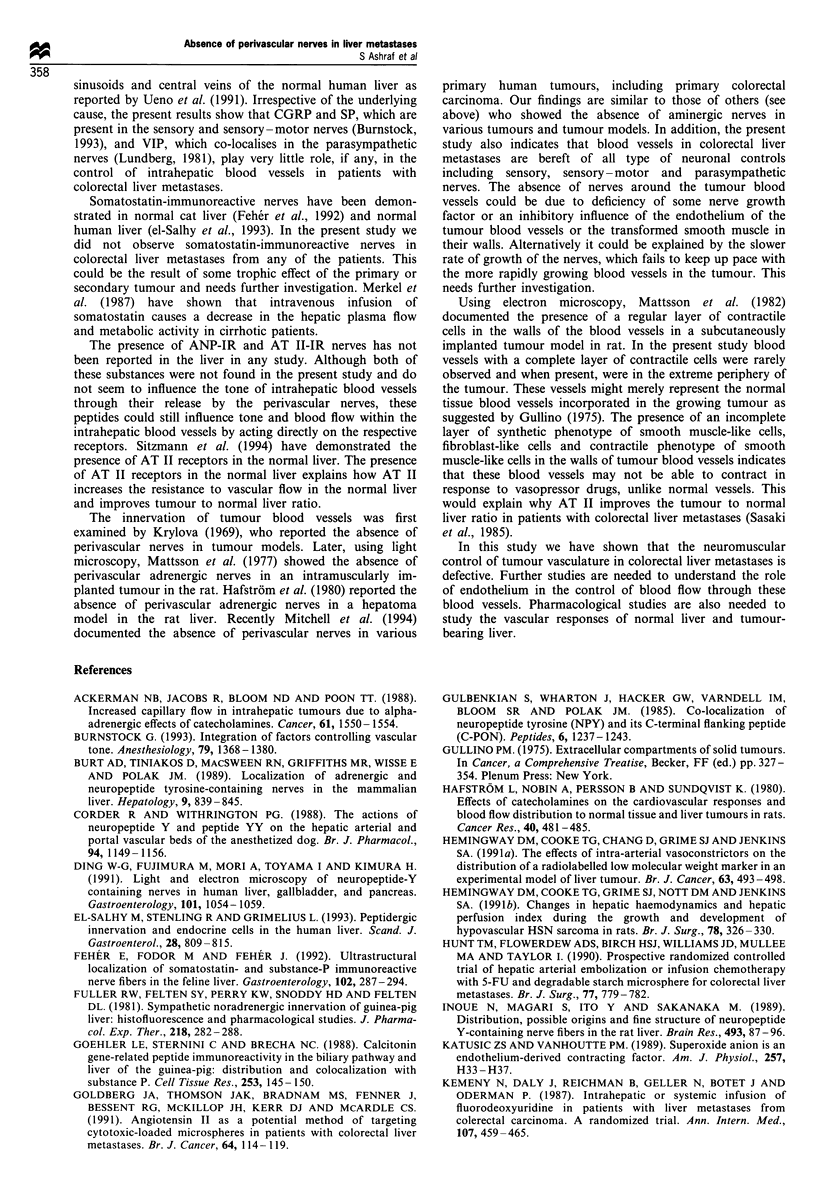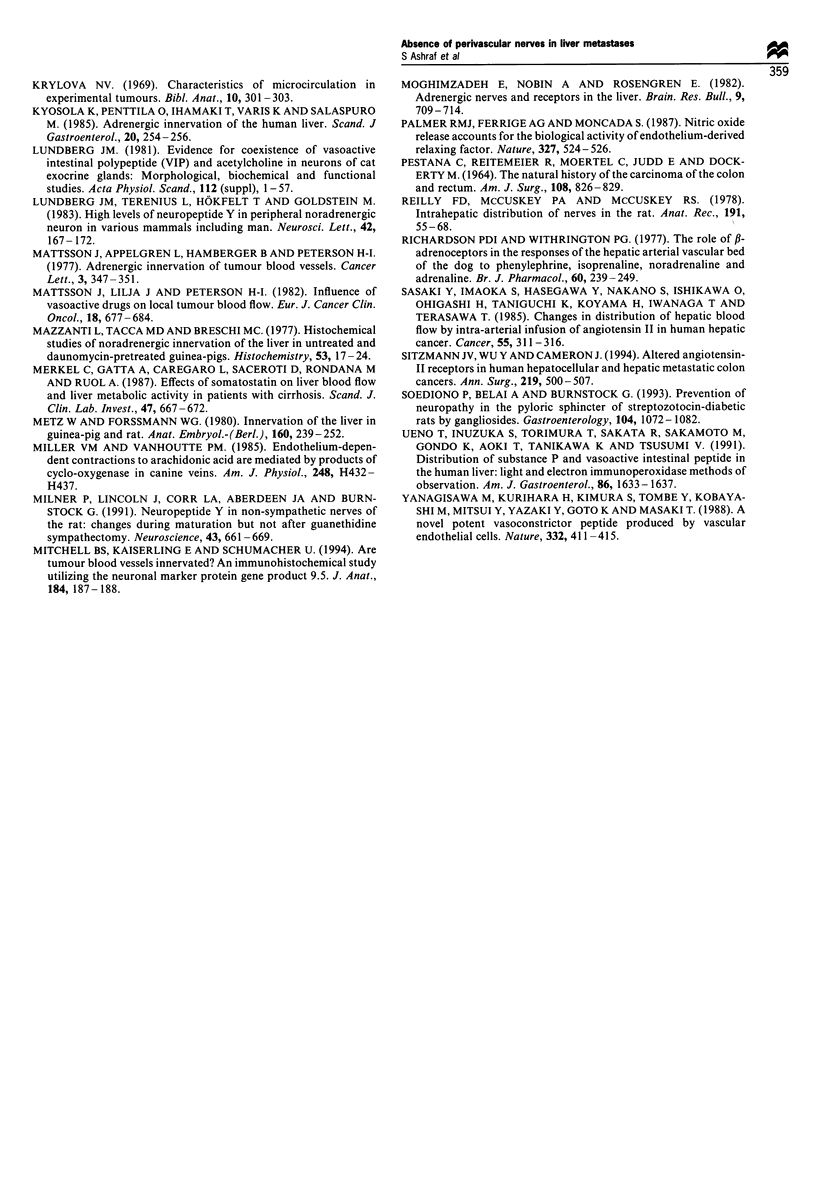# The absence of autonomic perivascular nerves in human colorectal liver metastases.

**DOI:** 10.1038/bjc.1996.60

**Published:** 1996-02

**Authors:** S. Ashraf, R. Crowe, M. C. Loizidou, M. Turmaine, I. Taylor, G. Burnstock

**Affiliations:** Department of Anatomy and Developmental Biology, University College London, UK.

## Abstract

**Images:**


British Journal of Cancer (1996) 73, 349-359
© 1996 Stockton Press All rights reserved 0007-0920/96 $12.00

### The absence of autonomic perivascular nerves in human colorectal liver metastases

S Ashraf[1], R Crowe[1], M C Loizidou[2], M Turmaine[1], I Taylor[2] and G Burnstock[1]

[1]*Department of Anatomy and Developmental Biology, University College London, Gower Street, London WC1E 6BT, UK;* [2]*Department of Surgery, University College London, 67-73 Riding House Street, London W1P 7LD, UK.*

**Summary** The peptidergic/aminergic innervation of normal liver and tumour blood vessels was investigated in order to determine vascular control with a view to improving the efficacy of hepatic arterial cytotoxic infusion in the treatment of colorectal liver metastases. Selected areas of liver metastases and macroscopically normal liver from resection specimens ($n = 13$) were studied using light microscope immunohistochemistry for the presence of protein gene product 9.5 (PGP), vasoactive intestinal polypeptide (VIP), neuropeptide Y (NPY), calcitonin gene-related peptide (CGRP), substance P (SP) and tyrosine hydroxylase (TH). The ultrastructure of blood vessels supplying liver metastases and their perivascular innervation were also examined by transmission electron microscopy. In the normal liver, perivascular immunoreactive nerve fibres containing PGP, NPY and TH were observed around the interlobular blood vessels and along the sinusoids and the central vein of the hepatic lobule. The greatest density of immunoreactive nerve fibres was seen for PGP, followed (in decreasing order) by NPY and TH. VIP, SP and CGRP immunoreactivity was observed only in nerve bundles associated with the large interlobular blood vessels. In contrast, no perivascular immunoreactive nerves were observed in colorectal liver metastases. Electron microscopy confirmed the absence of perivascular nerves in liver metastases. In addition, it showed that the walls of these blood vessels were composed of a layer of endothelial cells surrounded by an incomplete or, very rarely in the periphery of the tumour, a complete, layer of synthetic phenotype of smooth muscle-like cells. These results imply that the blood vessels supplying liver metastases are bereft of normal neuronal regulation; whether there is a role for endothelial cell control of blood flow in these vessels is not yet known.

**Keywords:** liver; metastases; blood vessels; nerves

Colorectal cancer is the second most prevalent cancer accounting for about 19 000 deaths in the UK each year. The liver is the most common site of colorectal metastases; the latter occur in about 70% of patients at post mortem (Pestana *et al.*, 1964) and account for up to 50% of all colorectal cancer deaths.

Surgical resection offers the only hope of cure, but in the majority of these patients, when multiple metastases are present, this is not feasible. In addition, the results of many available adjuvant therapies have been disappointing. Hepatic artery infusion (HAI) chemotherapy has, however, shown some promise and is associated with an increased tumour response rate compared with systemic chemotherapy (Kemeny *et al.*, 1987). A randomised trial has suggested that HAI chemotherapy improves the survival rate compared with untreated controls (Hunt *et al.*, 1990). There is some evidence that co-administration of vasoactive agents such as angiotensin II (AT II) can improve the efficacy of HAI chemotherapy (Goldberg *et al.*, 1991; Hemingway *et al.*, 1991*a*). However, Sasaki *et al.* (1985) have shown that AT II reaches the peak of its action at about 100 s from the start of the infusion. After this time there is a gradual fall in the tumour to normal liver ratio despite its continuous infusion for 3–4 min. This short duration of action limits the efficacy of AT II. In contrast with the empirical use of AT II, vascular manipulation might be achieved by administration of vasoactive substances, normally present in the liver but not in the tumour, e.g. various amines and peptides that are present in the perivascular nerves of the normal liver (Gulbenkian *et al.*, 1985; Goehler *et al.*, 1988; Ueno *et al.*, 1991).

It has been shown in different mammalian species that the neuronal control of the normal liver and its vasculature uses not only noradrenaline but also various peptides; the latter include substance P (SP) (Ueno *et al.*, 1991; Fehér *et al.*, 1992), vasoactive intestinal polypeptide (VIP) (Ueno *et al.*, 1991), calcitonin gene-related peptide (CGRP) (Goehler *et al.*, 1988), somatostatin (SOM) (Fehér *et al.*, 1992), neuropeptide Y (NPY) (Gulbenkian *et al.*, 1985; Inoue *et al.*, 1989; Ding *et al.*, 1991); tyrosine hydroxylase (TH) can be used as a marker for sympathetic nerves (Burt *et al.*, 1989).

The purpose of the present study is to investigate the perivascular innervation of human colorectal liver metastases compared with that of the normal liver, with the objective of improving the efficacy of intrahepatic arterial therapy.

Correspondence: G Burnstock
Received 21 April 1995; revised 17 August 1995; accepted 11 September 1995

### Material and methods

*Patients and tissue specimens*

13 patients undergoing surgery for colorectal liver metastases were included in the study. The surgical specimens consisted of metastases and normal liver some distance from the tumour. It was possible to take specimens of both the normal liver and the tumour from seven patients (one of these was used solely for electron microscopy). Tumour alone was obtained from four patients (including two peritoneal metastases from one patient) and normal liver alone from two patients.

*Immunohistochemistry*

Tissue blocks were fixed in 4% paraformaldehyde in phosphate-buffered saline (PBS) for 3 h at 4°C and then washed in 7% sucrose in PBS containing 0.01% sodium azide and stored at 4°C for at least 18 h. The tissue was then mounted in Tissue Tek (OCT; Miles, Elkhart, IN, USA) and 10 μm sections were cut on a cryostat (Reichert-Jung, Cambridge Instruments, Germany) at −25°C. The sections were mounted on gelatine-coated slides and incubated in humid chambers at room temperature for 18 h with polyclonal antisera to general neuronal marker protein gene product 9.5 (PGP) (Ultraclone, Isle of Wight, UK), TH (Affiniti, Nottingham, UK) and to the following neuropeptides: VIP (INC, Berkshire, UK), NPY (Peninsula Laboratories, St. Helens, UK), CGRP (Cambridge Research

Laboratories, UK), SP (Cambridge Research Laboratories), AT II (Peninsula Laboratories), SOM (Affiniti) and atrial natriuretic peptide (ANP) (Cambridge Research Laboratories) at dilutions of 1 : 1000. The preparation was washed in PBS and incubated with biotin-conjugated goat anti-rabbit immunoglobin at a dilution of 1 : 250 for 1 h, washed in PBS and incubated with streptavidin–fluorescein isothiocyanate conjugate at 1 : 100 dilution, for a further hour. The sections were incubated with 0.1% pontamine sky blue (BDH, UK) in 1% dimethylsulphoxide (DMSO) (Sigma, UK) in PBS to reduce background autofluorescence. Specimens of the tumour and normal liver were processed simultaneously under identical conditions. Immunoreactivity was viewed using a Zeiss microscope equipped with a KP560 filter for viewing FITC fluorescence. Selected areas were photographed on Kodak TMX 3200 film. Phase-contrast photographs of the liver parenchyma were also taken in order to localise immunoreactivity in relation to intralobular structures.

For controls, the tissues were incubated with antibody inactivated by the addition of excess antigen (10 nmol of antigen to 1 ml of corresponding antiserum) or by using normal rabbit serum as a first layer.

Sections were also stained with haematoxylin and eosin (H & E) and van Geison's stains for histological examination of the tissues.

### *Image analysis*

The Seescan image processing system (Seescan Imaging, Cambridge, UK) was used to assess the density of PGP-immunoreactive (IR), NPY-IR and TH-IR nerve fibres in the liver parenchyma as described by Soediono *et al*, (1993). Briefly, using a 10 × objective, ten representative areas of the liver parenchyma were computer analysed for each type of immunoreactive nerve i.e. PGP-IR, NPY-IR and TH-IR nerves in a constant area ($2.557 \times 10^5\ \mu m^2 \pm 7.02 \times 10^3$) of the parenchyma to reduce the field selection errors.

The computer-assisted image analysis converted the image into a binary form corresponding to positive or negative immunofluorescence. A threshold was set to remove background labelling, and the resultant fluorescent area was represented as a percentage of the whole area of the frame.

### *Statistical analysis*

The results are given as mean ± s.e.m. The results for each group of immunofluorescent nerves, in any patient, were tested against each of the remaining two groups using the Mann–Whitney *U*-test. A level of probability of 0.05 or less was considered to be significant. The means of the density of immunofluorescence of each type of intraparenchymal nerves in the normal liver were compared with those of the tumour using the Mann–Whitney *U*-test.

### *Electron microscopy*

The tissue was immerse fixed in a mixture of 2% paraformaldehyde and 2% glutaraldehyde in 0.1 M phosphate buffer (pH 7.4). The tissue was then divided in 1 mm$^3$ blocks and left in the same fixative overnight at 4°C. After

**Figure 1** Histology of normal human liver and colorectal liver metastases. (**a**) A liver lobule and portal tract (PT) (stained with H & E). The following structures are observed in the lobule; central vein (CV), sinusoids (S) and hepatic cords (H). Calibration bar = 100 μm. (**b**) Microscopic structure of the colorectal liver metastases (toluidine blue staining). Note that the gland alveoli (A) are surrounded by fibrous stroma (S). Calibration bar = 50 μm.

**Figure 2** Microscopic structure of colorectal liver metastases. (**a**) Note that there are no NPY-IR nerves in the colorectal liver metastases. Some autofluorescent areas are observed (A). These are more prominent with PGP than with NPY. These stained pink with van Geison's (not shown), which is specific for collagen. Calibration bar = 80 μm. (**b**) Light microscopic structure of colorectal liver metastases. (toluidine blue stain) showing a blood vessel (BV) in the vicinity of the gland alveoli (A). Calibration bar = 50 μm.

osmication (1 h in 1% osmium tetroxide with 0.1 M phosphate buffer) the sections were stained for 45 min in 2% uranyl acetate in water at 4°C, dehydrated in ethanols, cleared in propylene oxide and embedded in Araldite. Semithin sections (1 $\mu$m) were cut with glass knives, and stained with toluidine blue. From the adjacent area ultrathin sections (60–70 nm) were cut using a diamond knife on Reichert Ultracut ultramicrotome and collected on mesh grids and single-slot grids coated with thin Formvar film. These sections were counterstained with lead citrate and viewed with a Joel electron microscope.

### Results

### *Light microscopy – histology*

*Normal liver* In the normal liver, hexagonal liver lobules with portal tracts occupying the corners of the hepatic lobule were observed. Within the lobule, hepatic cords were separated from each other by the sinusoids. In the centre of the hepatic lobule the central vein was observed (Figure 1a). Larger interlobular branches of the hepatic artery and the portal vein were observed in the interlobular connective tissue. There was no evidence of micrometastases.

**Figure 3** Distribution of immunoreactive nerves around interlobular branch of the hepatic artery. (**a**) PGP-IR nerves around interlobular branch of the hepatic artery. Note that the nerves are distributed at the adventitial–medial border (NF). In addition large nerve bundles (NB) are present in the adventitia of these blood vessels. Note the autofluorescence in internal elastic lamina (I). (**b**) NPY-IR nerves around interlobular branch of the hepatic artery. Note that immunoreactive nerves are present both at the adventitial–medial border (NF) and in the adventitia. Note the autofluorescence in internal elastic lamina (I). (**c**) VIP-IR nerves around interlobular branch of the hepatic artery. Note that immunoreactive nerves (N) are present only in the adventitial nerve bundles. They are not present at the adventitial–medial border of the blood vessel. Note the autofluorescence in the internal elastic lamina (I). (**d**) Distribution of TH-IR nerves around the interlobular branch of the hepatic artery. Note that the nerves are present both at the adventitial–medial border (NF) and in the adventitia (NB). Note the autofluorescence in the internal elastic lamina (I). Calibration bar = 80 $\mu$m.

*Liver metastases* Histopathological (H & E) examination demonstrated that 12 out of 13 patients had moderately differentiated adenocarcinoma (Figure 1b). The 13th patient had a poorly differentiated adenocarcinoma. The amount of stroma varied in different regions of the tumour. It consisted of bundles of collagen fibres as it stained pink with van Geison's. Tumour blood vessels were observed in the stroma of the tumour (Figure 2b).

*Immunohistochemistry*

*Normal liver* PGP-IR nerves (Figure 3a) were observed as single varicose nerve fibres or thicker fascicles at the adventitial medial border of the interlobular branches of the hepatic artery (150–1500 μm) and the portal vein in the interlobular connective tissue. In the adventitia of these blood vessels thick nerve bundles were also observed.

**Figure 4** Distribution of nerves within the liver parenchyma on fluorescent microscopy (**a**, **c** and **e**) with phase contrast pictures (**b**, **d** and **f**) of the same field. (**a**) PGP-IR nerves (N) are observed along the sinusoids (S) and hepatic cords (H). (**b**) Phase contrast photograph of the same field as in (**a**) showing sinusoids (S) and hepatic cords (H). (**c**) NPY-IR (N) nerves in the liver parenchyma. Note that the nerves run along the sinusoids (S) and the hepatic cords (H). (**d**) Phase contrast photograph of the same field as shown in (**c**). Note the hepatic cords (H) and the sinusoids (S). (**e**) TH-IR nerves (N) in the liver parenchyma. Note that the nerves run along the sinusoids (S) and the hepatic cords (H). (**f**) Phase contrast photograph of the same field as in (**e**) showing sinusoids (S) and hepatic cords (H). Calibration bar = 125 μm.

Smaller interlobular branches (less than 150 μm in diameter) of hepatic artery and portal vein, of the portal tract, had single nerve fibre or thicker fascicles associated with them. No large bundles were observed at this site. All divisions of the arteries were more densely innervated than the corresponding veins. A few nerves were also observed in association with the interlobular bile ducts.

In the liver lobule PGP-IR varicose nerve fibres were observed along the sinusoids and the hepatic cords (Figure 4a). Outer portions of the liver lobules were more densely innervated than the central portions. Occasionally these nerves were seen to traverse to the central vein of the hepatic lobule. Although the pattern of innervation was the same throughout the liver, some lobules were more densely innervated than the others in any given section. In addition to these variations, liver parenchyma in some patients was found to be more densely innervated than in other patients.

NPY-IR and TH-IR nerves had similar patterns of distribution both around interlobular blood vessels and within the hepatic lobule (Figures 3b and d, 4c and e). The greatest density of IR nerves was observed for PGP followed, in decreasing order, by those of NPY-IR and TH-IR nerves (Table I). Statistical analysis of the data showed that in any given patient the density of PGP-IR nerves was significantly more than NPY-IR nerves ($P<0.035$) and TH-IR nerves ($P<0.0001$). Similarly, the density of NPY-IR nerves was significantly more than that of TH-IR nerves ($P<0.002$).

VIP-IR, SP-IR and CGRP-IR nerve fibres were not observed except for a few IR nerve fibres in the thick nerve bundles running in the adventitia of the larger interlobular blood vessels (Figure 3c). AT II-IR, SOM-IR and ANP-IR nerves were absent.

*Liver metastases* No neuronal immunoreactivity (Figure 2a) was observed in association with the blood vessels or parenchyma in any of the colorectal liver or peritoneal metastases (Table II). However, in the stroma of the colorectal liver metastases thick bundles of autofluorescent tissue were observed. This immunoreactive tissue stained pink with van Geison's which is specific for collagen.

### *Electron microscopy*

*Normal liver* In the portal tract, the branches of the hepatic artery and the portal vein were observed. The walls of these blood vessels were composed of a layer of endothelial cells surrounded by a layer of contractile phenotype of smooth muscle cells. Bundles of unmyelinated nerve fibres were observed in the connective tissue around blood vessels (Figure 5a). The nerve fibres of these bundles contained variable sized dense-cored (600–1300° A diameter) and clear vesicles (250–800° A diameter). The nerves were more dense around the branches of the hepatic artery than with those of the portal vein. Occasionally they were observed in association with the interlobular bile ducts.

In the liver lobule, nerve fibres were observed in the space of Disse running close to the hepatocytes. The latter showed invaginations of the surface membrane where they made contact with the nerve varicosity (Figure 5b and c). The diameter of these nerve varicosities ranged from 650 nm to 2000 nm and they contained dense-cored and clear vesicles of variable size. On occasion, these nerve varicosities were observed in the proximity of the sinusoidal endothelial cells, Ito cells or Kupffer cells.

*Liver metastases* Colorectal liver metastases showed marked heterogeneity of blood vessels, both regarding their size and density of distribution. The centre of the tumour showed loss of boundaries of all types of cells. Numerous free red blood cells were observed in this region.

More peripherally, blood vessels of variable size were observed. Their size ranged from 8–55 μm (Figure 6a and b). Irrespective of size, the blood vessels were remarkably similar in structure. The wall of these blood vessels was made up of a single layer of endothelial cells resting on a basement membrane. Occasionally these blood vessels were surrounded by an incomplete layer of smooth muscle cells of variable phenotype, nearly always of the secretory (proliferative) form (Figure 7a). Very rarely, in the extreme periphery of the tumour, blood vessels with a complete layer of contractile phenotype smooth muscle cells were observed (Figure 7b).

The endothelial cells had occasional cytoplasmic fenestrations, a large amount of rough endoplasmic reticulum (RER), Golgi apparatus and numerous free ribosomes and occasional pinocytic vesicles. Weibel–Palade bodies were not observed (Figure 8a). In summary, the various types of cells observed in the walls of tumour blood vessels were as follows:

(i) Fibroblast-like cells: these had an elongated nucleus and one or two nucleoli. Their cytoplasm showed abundant RER, Golgi apparatus and mitochondria in the vicinity of the nucleus. Occasional myofilaments were seen especially under the cell membrane. Occasionally caveolae were also observed (Figure 6a and b).

(ii) Synthetic (proliferative) phenotype of smooth muscle-like cells: these cells contained dense bands and cytoplasmic dense bodies, a large number of caveolae and abundant RER, Golgi apparatus and some myofilaments (Figures 7a and b, 8b).

(iii) Contractile phenotype of smooth muscle-like cells: these contained large numbers of caveolae, dense bands, cytoplasmic dense bodies and myofilaments. Small amounts of RER

**Table I** The density of immunofluorescence for different peptides in the histologically normal areas of the livers of the patients with colorectal liver metastases (mean ± s.e.m)

| *Sr no.*[a] | *PGP* | *NPY* | *TH* |
|---|---|---|---|
| 1 | 1.59±0.33 | 1.28±0.37 (80.5)[b] | – |
| 2 | 1.32±0.37 | 0.71±0.29 (53.8) | 0.33±0.05 (25.0) |
| 3 | 3.92±0.95 | 1.69±0.48 (43.1) | 0.69±0.20 (17.6) |
| 4 | 1.39±0.33 | 1.02±0.29 (73.4) | – |
| 5 | 1.18±0.37 | 0.48±0.23 (40.7) | – |
| 6 | 2.57±0.79 | 1.59±0.36 (61.7) | 0.23±0.09 (8.9) |
| 7 | 0.66±0.15 | – | – |
| 8 | 2.16±0.67 | 0.78±0.29 (36.1) – | – |
| Mean ± s.e.m. | 1.85±0.36 | 1.08±0.17 (58.4) | 0.42±0.14 (22.7) |

[a]Specimens of normal liver, obtained from eight patients, were subjected to immunocytochemistry. A further specimen of normal liver was used only for electron microscopy. In the remaining four patients a specimen of only the tumour was obtained. Figures represent the mean percentage (%) area of fluorescence in ten frames. [b]Figures in brackets represent the proportion of immunoreactive nerve fibres as a percentage of PGP-IR nerves. (–), Computerised analysis of the image was not possible in these cases because of the high background and poor contrast.

**Table II** Comparison of the densities of different types of immunoreactive perivascular nerves in the parenchyma of the normal liver and colorectal liver metastases (mean ± s.e.m.)

| *Nerve population* | *Normal* | *Tumour* | *Normal* vs *tumour*[a] (P-*value*) |
|---|---|---|---|
| PGP | 1.85±0.36 | 0 | $P=0.0003$ |
| NPY | 1.08±0.17 (58.4)[b] | 0 | $P=0.0003$ |
| TH | 0.42±0.14 (22.7) | 0 | $P=0.008$ |

[a]The density of innervation was compared using the Mann–Whitney *U*-test. [b]Figures represent the mean of the percentage (%) area of fluorescence for all patients. Figures in brackets represent the proportion of immunoreactive nerve fibres as a percentage of PGP-IR nerves.

**Figure 5** Ultrastructure of blood vessels and distribution of perivascular nerves in the normal liver. (**a**) A bundle of unmyelinated nerve fibres in the vicinity of an arteriole of the portal tract. Note the lumen (L) containing red blood cell (RBC), endothelial cell (E), smooth muscle cell of contractile phenotype (SMC) and nerve bundle (NB). Calibration bar = 1.0 μm. (**b**) A bundle of unmyelinated nerve fibres (N) in the space of Disse (SD). Note the hepatocyte (H), sinusoid (S) and the sinusoidal endothelial cell (E). Calibration bar = 1.0 μm. (**c**) High-power photograph of the nerve profile observed in figure (**b**) showing nerve fibres (N) in the space of Disse (SD). Note the hepatocyte (H), sinusoid (S) and sinusoidal endothelial cell (E). Calibration bar = 1.0 μm.

and Golgi appararus were also observed. Their basement membrane was continuous (Figure 8a). They were longer cells so that sections through the nucleus were rare compared with the synthetic phenotype cells.

Perivascular connective tissue was composed of bundles of collagen fibres. No perivascular nerves were observed in any of the blood vessels.

### Discussion

The control of vascular tone involves both nerves and the endothelium (Burnstock, 1993). The nerves around the blood vessels can be divided into perivascular nerves and paravascular nerves. The perivascular nerves form a fine plexus of nerves around the tunica media of the blood vessel, i.e. at the adventitial–medial border and are responsible for the control of tone of that blood vessel. The paravascular nerves are arranged in larger nerve bundles that run in the adventitia of the blood vessels and are destined to the vascular and non-vascular structures further along that blood vessel. The vascular endothelium can effect vascular tone by endothelial-derived relaxing factors (EDRFs) (Palmer *et al.*, 1987) and endothelial–derived constricting factors (EDCFs) (Miller and Vanhoutte, 1985; Katusic and Vanhoutte, 1989; Yanagisawa *et al.*, 1988).

The present study has shown that in the normal liver, PGP-IR, NPY-IR and TH-IR nerves are present at the perivascular and paravascular sites around the larger intrahepatic blood vessels and along the intraparenchymal blood vessels, whereas SP-IR, VIP-IR and CGRP-IR nerves are present only in the paravascular nerve bundles running along larger intrahepatic blood vessels. AT II-IR, SOM-IR and ANP-IR nerves are absent. In contrast, blood vessels in colorectal liver metastases lack perivascular innervation. This finding is substantiated by transmission electron microscopy, which also demonstrated the absence of nerve profiles. Furthermore, except for an occasional blood vessel in the periphery of the tumour, contractile smooth muscle in the walls of most blood vessels was either incomplete or absent altogether.

The aminergic innervation of the normal liver has been examined in depth in various mammalian species (Mazzanti *et al.*, 1977; Fuller *et al.*, 1981; Moghimzadeh *et al.*, 1982) including man (Moghimzadeh *et al.*, 1982; Kyosola *et al.*, 1985). In the present study we only investigated the distribution of TH-IR nerves. TH is an enzyme used in the synthesis of the amines such as noradrenaline and adrenaline. The distribution of TH-IR perivascular nerves around the interlobular blood vessels and within the liver parenchyma is in concordance with the aminergic innervation of the normal liver as reported in earlier studies (see above). It has been shown in hepatic arterial vascular bed of dog that noradrenaline increases hepatic arterial vascular resistance and decreases blood flow (Richardson and Withrington, 1977). This pharmacological property has been shown to be useful in improving the tumour to normal liver ratio in rat (Hafström *et al.*, 1980; Ackerman *et al.*, 1988). However, Ackerman *et al.* (1988) have shown that after an intraportal injection of noradrenaline, tumour to normal liver ratio improved for only 134 s in Sprague–Dawley rats with liver tumours. After this time a baseline level of blood flow was reached in the liver tumours. This short duration of action limits the use of noradrenaline as a vasopressor for targeting hepatic artery infusion chemotherapy.

The presence of NPY, which co-exists with noradrenaline in sympathetic nerves (Lundberg *et al.*, 1983), has been reported in the perivascular nerves of liver in rat (Gulbenkian *et al.*, 1985; Inoue *et al.*, 1989). In contrast to the present study, these workers have reported the absence of intraparenchymal nerves along the sinusoids. This could be explained by the difference in species; the absence of perivascular nerves in the liver parenchyma of the rat has been reported in many published studies (Reilly *et al.*, 1978; Metz and Forssmann, 1980; Inoue *et al.*, 1989). Widespread distribution of NPY-IR and TH-IR nerves within the normal liver indicates that most of the intrahepatic nerves are sympathetic in type. It has been shown that in addition to its inherent vasoconstrictor action, NPY augments the vasoconstrictive actions of noradrenaline on various vessels, including the hepatic artery (Corder and Withrington, 1988). It is, therefore, a potential candidate to be used in combination with noradrenaline to achieve the desirable vascular effects to enhance the efficacy of HAI chemotherapy. By enhancing the actions of noradrenaline, NPY can, at least theoretically, improve vasoconstriction in the normal liver and divert blood flow to the tumour tissue and thus achieve the improved tumour to normal liver ratio for a longer duration compared with noradrenaline alone. This needs further investigation.

In the present study, the density of NPY-IR nerves was

**Figure 6** Ultrastructure of blood vessels in colorectal liver metastases. (**a**) A small capillary 8 $\mu$m in diameter with red blood cell (RBC) in the lumen surrounded by endothelial cell (E). Note the fibroblast-like cell (F) surrounding the endothelial cells. Calibration bar = 1.0 $\mu$m. (**b**) A large thin-walled blood vessel with a wide lumen (55 $\mu$m in diameter) showing the red blood cells (RBC) in the lumen, endothelial cell (E), and the fibroblast-like cell (F) in the perivascular connective tissue. Note that there are no perivascular nerves. Calibration bar = 10 $\mu$m.

**Figure 7** Ultrastructure of blood vessels in colorectal liver metastases. (**a**) A blood vessel with an incomplete layer of smooth muscle cells of synthetic (proliferative) phenotype in its wall. Note the lumen (L), endothelial cells (E) and synthetic phenotype of smooth muscle-like cell (SMC). Calibration bar = 2.0 μm. (**b**) A blood vessel with a layer of synthetic phenotype of smooth muscle-like cells forming complete layer in the wall. Blood vessels with this structure are confined to the extreme periphery of the tumour and are observed very occasionally. Note the lumen (L), endothelial cells (E) and synthetic phenotype of smooth muscle-like cell (SMC). Calibration bar = 2.0 μm.

significantly greater than that of TH-IR nerves. This difference may be due to the fact that TH is a neurotransmitter-synthesising enzyme and therefore, may be present at a lower concentration within nerves than a neurotransmitter. However, it is also possible that the higher density of NPY-IR nerves may represent (in part) a separate population of non-sympathetic nerves as seen in the rat urinary bladder, vas deferens, mesenteric vein and superior cervical ganglion (Milner *et al.*, 1991). Goehler *et al.* (1988) have reported the existence of CGRP-IR and SP-IR nerves around interlobular blood vessels and within the lobules of the guinea pig liver. These are likely to represent sensory-motor nerves (Burnstock, 1993). However, in our study, we did not observe any intraparenchymal CGRP-IR or SP-IR nerves in the human liver. This could be due to a difference of species, antibodies or immunocytochemical technique. Alternatively, the reduction of these vasodilator peptides in a histologically normal area of a liver harbouring metastases from colorectal cancer could be due to some trophic effect of either the primary tumour or the metastasis itself and may explain the increased splanchnic and portal vascular resistances in the animal model of colorectal liver metastases as reported by Hemingway *et al.* (1991*b*). This would also explain the scant presence of VIP-immunoreactive nerves in the normal liver, as observed in the present study, contrary to their frequent occurrence around interlobular blood vessels,

**Figure 8** Ultrastructure of the endothelial cell and the synthetic phenotype of smooth muscle cell in the wall of a blood vessel of colorectal liver metastases. (**a**) Endothelial cell (E) of a blood vessel in the colorectal liver metastasis. Note the smooth muscle cell (SMC) in the wall of the blood vessel showing caveolae (C), dense bands (DB) and cytoplasmic dense bodies (D). Calibration bar = 0.5 $\mu$m. (**b**) Synthetic phenotype of smooth muscle-like cell. Note the rough endoplasmic reticulum (RER) in the perinuclear region of the cytoplasm, dense bands (DB), cytoplasmic dense bodies (D), and caveolae (C). An artifact (A) is created by the dissolved-out glycogen during tissue processing. Calibration bar = 1.0 $\mu$m.

sinusoids and central veins of the normal human liver as reported by Ueno *et al.* (1991). Irrespective of the underlying cause, the present results show that CGRP and SP, which are present in the sensory and sensory–motor nerves (Burnstock, 1993), and VIP, which co-localises in the parasympathetic nerves (Lundberg, 1981), play very little role, if any, in the control of intrahepatic blood vessels in patients with colorectal liver metastases.

Somatostatin-immunoreactive nerves have been demonstrated in normal cat liver (Fehér *et al.*, 1992) and normal human liver (el-Salhy *et al.*, 1993). In the present study we did not observe somatostatin-immunoreactive nerves in colorectal liver metastases from any of the patients. This could be the result of some trophic effect of the primary or secondary tumour and needs further investigation. Merkel *et al.* (1987) have shown that intravenous infusion of somatostatin causes a decrease in the hepatic plasma flow and metabolic activity in cirrhotic patients.

The presence of ANP-IR and AT II-IR nerves has not been reported in the liver in any study. Although both of these substances were not found in the present study and do not seem to influence the tone of intrahepatic blood vessels through their release by the perivascular nerves, these peptides could still influence tone and blood flow within the intrahepatic blood vessels by acting directly on the respective receptors. Sitzmann *et al.* (1994) have demonstrated the presence of AT II receptors in the normal liver. The presence of AT II receptors in the normal liver explains how AT II increases the resistance to vascular flow in the normal liver and improves tumour to normal liver ratio.

The innervation of tumour blood vessels was first examined by Krylova (1969), who reported the absence of perivascular nerves in tumour models. Later, using light microscopy, Mattsson *et al.* (1977) showed the absence of perivascular adrenergic nerves in an intramuscularly implanted tumour in the rat. Hafström *et al.* (1980) reported the absence of perivascular adrenergic nerves in a hepatoma model in the rat liver. Recently Mitchell *et al.* (1994) documented the absence of perivascular nerves in various primary human tumours, including primary colorectal carcinoma. Our findings are similar to those of others (see above) who showed the absence of aminergic nerves in various tumours and tumour models. In addition, the present study also indicates that blood vessels in colorectal liver metastases are bereft of all type of neuronal controls including sensory, sensory–motor and parasympathetic nerves. The absence of nerves around the tumour blood vessels could be due to deficiency of some nerve growth factor or an inhibitory influence of the endothelium of the tumour blood vessels or the transformed smooth muscle in their walls. Alternatively it could be explained by the slower rate of growth of the nerves, which fails to keep up pace with the more rapidly growing blood vessels in the tumour. This needs further investigation.

Using electron microscopy, Mattsson *et al.* (1982) documented the presence of a regular layer of contractile cells in the walls of the blood vessels in a subcutaneously implanted tumour model in rat. In the present study blood vessels with a complete layer of contractile cells were rarely observed and when present, were in the extreme periphery of the tumour. These vessels might merely represent the normal tissue blood vessels incorporated in the growing tumour as suggested by Gullino (1975). The presence of an incomplete layer of synthetic phenotype of smooth muscle-like cells, fibroblast-like cells and contractile phenotype of smooth muscle-like cells in the walls of tumour blood vessels indicates that these blood vessels may not be able to contract in response to vasopressor drugs, unlike normal vessels. This would explain why AT II improves the tumour to normal liver ratio in patients with colorectal liver metastases (Sasaki *et al.*, 1985).

In this study we have shown that the neuromuscular control of tumour vasculature in colorectal liver metastases is defective. Further studies are needed to understand the role of endothelium in the control of blood flow through these blood vessels. Pharmacological studies are also needed to study the vascular responses of normal liver and tumour-bearing liver.

### References

ACKERMAN NB, JACOBS R, BLOOM ND AND POON TT. (1988). Increased capillary flow in intrahepatic tumours due to alpha-adrenergic effects of catecholamines. *Cancer*, **61**, 1550–1554.

BURNSTOCK G. (1993). Integration of factors controlling vascular tone. *Anesthesiology*, **79**, 1368–1380.

BURT AD, TINIAKOS D, MACSWEEN RN, GRIFFITHS MR, WISSE E AND POLAK JM. (1989). Localization of adrenergic and neuropeptide tyrosine-containing nerves in the mammalian liver. *Hepatology*, **9**, 839–845.

CORDER R AND WITHRINGTON PG. (1988). The actions of neuropeptide Y and peptide YY on the hepatic arterial and portal vascular beds of the anesthetized dog. *Br. J. Pharmacol.*, **94**, 1149–1156.

DING W-G, FUJIMURA M, MORI A, TOYAMA I AND KIMURA H. (1991). Light and electron microscopy of neuropeptide-Y containing nerves in human liver, gallbladder, and pancreas. *Gastroenterology*, **101**, 1054–1059.

EL-SALHY M, STENLING R AND GRIMELIUS L. (1993). Peptidergic innervation and endocrine cells in the human liver. *Scand. J. Gastroenterol.*, **28**, 809–815.

FEHÉR E, FODOR M AND FEHÉR J. (1992). Ultrastructural localization of somatostatin- and substance-P immunoreactive nerve fibers in the feline liver. *Gastroenterology*, **102**, 287–294.

FULLER RW, FELTEN SY, PERRY KW, SNODDY HD AND FELTEN DL. (1981). Sympathetic noradrenergic innervation of guinea-pig liver: histofluorescence and pharmacological studies. *J. Pharmacol. Exp. Ther.*, **218**, 282–288.

GOEHLER LE, STERNINI C AND BRECHA NC. (1988). Calcitonin gene-related peptide immunoreactivity in the biliary pathway and liver of the guinea-pig: distribution and colocalization with substance P. *Cell Tissue Res.*, **253**, 145–150.

GOLDBERG JA, THOMSON JAK, BRADNAM MS, FENNER J, BESSENT RG, MCKILLOP JH, KERR DJ AND MCARDLE CS. (1991). Angiotensin II as a potential method of targeting cytotoxic-loaded microspheres in patients with colorectal liver metastases. *Br. J. Cancer*, **64**, 114–119.

GULBENKIAN S, WHARTON J, HACKER GW, VARNDELL IM, BLOOM SR AND POLAK JM. (1985). Co-localization of neuropeptide tyrosine (NPY) and its C-terminal flanking peptide (C-PON). *Peptides*, **6**, 1237–1243.

GULLINO PM. (1975). Extracellular compartments of solid tumours. In *Cancer, a Comprehensive Treatise*, Becker, FF (ed.) pp. 327–354. Plenum Press: New York.

HAFSTRÖM L, NOBIN A, PERSSON B AND SUNDQVIST K. (1980). Effects of catecholamines on the cardiovascular responses and blood flow distribution to normal tissue and liver tumours in rats. *Cancer Res.*, **40**, 481–485.

HEMINGWAY DM, COOKE TG, CHANG D, GRIME SJ AND JENKINS SA. (1991*a*). The effects of intra-arterial vasoconstrictors on the distribution of a radiolabelled low molecular weight marker in an experimental model of liver tumour. *Br. J. Cancer*, **63**, 493–498.

HEMINGWAY DM, COOKE TG, GRIME SJ, NOTT DM AND JENKINS SA. (1991*b*). Changes in hepatic haemodynamics and hepatic perfusion index during the growth and development of hypovascular HSN sarcoma in rats. *Br. J. Surg.*, **78**, 326–330.

HUNT TM, FLOWERDEW ADS, BIRCH HSJ, WILLIAMS JD, MULLEE MA AND TAYLOR I. (1990). Prospective randomized controlled trial of hepatic arterial embolization or infusion chemotherapy with 5-FU and degradable starch microsphere for colorectal liver metastases. *Br. J. Surg.*, **77**, 779–782.

INOUE N, MAGARI S, ITO Y AND SAKANAKA M. (1989). Distribution, possible origins and fine structure of neuropeptide Y-containing nerve fibers in the rat liver. *Brain Res.*, **493**, 87–96.

KATUSIC ZS AND VANHOUTTE PM. (1989). Superoxide anion is an endothelium-derived contracting factor. *Am. J. Physiol.*, **257**, H33–H37.

KEMENY N, DALY J, REICHMAN B, GELLER N, BOTET J AND ODERMAN P. (1987). Intrahepatic or systemic infusion of fluorodeoxyuridine in patients with liver metastases from colerectal carcinoma. A randomized trial. *Ann. Intern. Med.*, **107**, 459–465.

KRYLOVA NV. (1969). Characteristics of microcirculation in experimental tumours. *Bibl. Anat.*, **10**, 301–303.

KYOSOLA K, PENTTILA O, IHAMAKI T, VARIS K AND SALASPURO M. (1985). Adrenergic innervation of the human liver. *Scand. J Gastroenterol.*, **20**, 254–256.

LUNDBERG JM. (1981). Evidence for coexistence of vasoactive intestinal polypeptide (VIP) and acetylcholine in neurons of cat exocrine glands: Morphological, biochemical and functional studies. *Acta Physiol. Scand.*, **112** (suppl), 1–57.

LUNDBERG JM, TERENIUS L, HÖKFELT T AND GOLDSTEIN M. (1983). High levels of neuropeptide Y in peripheral noradrenergic neuron in various mammals including man. *Neurosci. Lett.*, **42**, 167–172.

MATTSSON J, APPELGREN L, HAMBERGER B AND PETERSON H-I. (1977). Adrenergic innervation of tumour blood vessels. *Cancer Lett.*, **3**, 347–351.

MATTSSON J, LILJA J AND PETERSON H-I. (1982). Influence of vasoactive drugs on local tumour blood flow. *Eur. J. Cancer Clin. Oncol.*, **18**, 677–684.

MAZZANTI L, TACCA MD AND BRESCHI MC. (1977). Histochemical studies of noradrenergic innervation of the liver in untreated and daunomycin-pretreated guinea-pigs. *Histochemistry*, **53**, 17–24.

MERKEL C, GATTA A, CAREGARO L, SACEROTI D, RONDANA M AND RUOL A. (1987). Effects of somatostatin on liver blood flow and liver metabolic activity in patients with cirrhosis. *Scand. J. Clin. Lab. Invest.*, **47**, 667–672.

METZ W AND FORSSMANN WG. (1980). Innervation of the liver in guinea-pig and rat. *Anat. Embryol.-(Berl.)*, **160**, 239–252.

MILLER VM AND VANHOUTTE PM. (1985). Endothelium-dependent contractions to arachidonic acid are mediated by products of cyclo-oxygenase in canine veins. *Am. J. Physiol.*, **248**, H432–H437.

MILNER P, LINCOLN J, CORR LA, ABERDEEN JA AND BURNSTOCK G. (1991). Neuropeptide Y in non-sympathetic nerves of the rat: changes during maturation but not after guanethidine sympathectomy. *Neuroscience*, **43**, 661–669.

MITCHELL BS, KAISERLING E AND SCHUMACHER U. (1994). Are tumour blood vessels innervated? An immunohistochemical study utilizing the neuronal marker protein gene product 9.5. *J. Anat.*, **184**, 187–188.

MOGHIMZADEH E, NOBIN A AND ROSENGREN E. (1982). Adrenergic nerves and receptors in the liver. *Brain. Res. Bull.*, **9**, 709–714.

PALMER RMJ, FERRIGE AG AND MONCADA S. (1987). Nitric oxide release accounts for the biological activity of endothelium-derived relaxing factor. *Nature*, **327**, 524–526.

PESTANA C, REITEMEIER R, MOERTEL C, JUDD E AND DOCKERTY M. (1964). The natural history of the carcinoma of the colon and rectum. *Am. J. Surg.*, **108**, 826–829.

REILLY FD, McCUSKEY PA AND McCUSKEY RS. (1978). Intrahepatic distribution of nerves in the rat. *Anat. Rec.*, **191**, 55–68.

RICHARDSON PDI AND WITHRINGTON PG. (1977). The role of $\beta$-adrenoceptors in the responses of the hepatic arterial vascular bed of the dog to phenylephrine, isoprenaline, noradrenaline and adrenaline. *Br. J. Pharmacol.*, **60**, 239–249.

SASAKI Y, IMAOKA S, HASEGAWA Y, NAKANO S, ISHIKAWA O, OHIGASHI H, TANIGUCHI K, KOYAMA H, IWANAGA T AND TERASAWA T. (1985). Changes in distribution of hepatic blood flow by intra-arterial infusion of angiotensin II in human hepatic cancer. *Cancer*, **55**, 311–316.

SITZMANN JV, WU Y AND CAMERON J. (1994). Altered angiotensin-II receptors in human hepatocellular and hepatic metastatic colon cancers. *Ann. Surg.*, **219**, 500–507.

SOEDIONO P, BELAI A AND BURNSTOCK G. (1993). Prevention of neuropathy in the pyloric sphincter of streptozotocin-diabetic rats by gangliosides. *Gastroenterology*, **104**, 1072–1082.

UENO T, INUZUKA S, TORIMURA T, SAKATA R, SAKAMOTO M, GONDO K, AOKI T, TANIKAWA K AND TSUSUMI V. (1991). Distribution of substance P and vasoactive intestinal peptide in the human liver: light and electron immunoperoxidase methods of observation. *Am. J. Gastroenterol.*, **86**, 1633–1637.

YANAGISAWA M, KURIHARA H, KIMURA S, TOMBE Y, KOBAYASHI M, MITSUI Y, YAZAKI Y, GOTO K AND MASAKI T. (1988). A novel potent vasoconstrictor peptide produced by vascular endothelial cells. *Nature*, **332**, 411–415.